# Oral Health Related Quality of Life and Xerostomia in Cardiovascular Patients: OHRQoL in Cardiac Patients

**DOI:** 10.31661/gmj.vi.3805

**Published:** 2025-08-08

**Authors:** Adel Tabesh, Hossein Karimzadeh, Narges Shahriari, Omid Fakheran Esfahani

**Affiliations:** ^1^ Department of Oral and Maxillofacial Medicine, Dental Research Center, Dental Research Institute, School of Dentistry, Isfahan University of Medical Sciences, Isfahan, Iran; ^2^ Dental Students Research Committee, School of Dentistry, Isfahan University of Medical Sciences, Isfahan, Iran; ^3^ Division of Oral Surgery and Orthodontics, Department of Dental Medicine and Oral Health, Medical University of Graz, Graz, Austria

**Keywords:** Oral Health, Quality of Life, Xerostomia, Cardiovascular

## Abstract

**Background:**

Cardiovascular patients frequently suffer from xerostomia and several modalities has been tried to alleviate the symptom. Improving Oral Health Related Quality of Life (OHRQoL) is a subjective index and considered as the final achievement in oral treatments. The aim of the present study was to evaluate OHRQoL in these patients, as well as its relation to xerostomia and some other factors.

**Materials and Methods:**

In this cross-sectional study, 244 patients participated. OHRQoL was assessed by Oral Health Impact Profile 14 (OHIP-14). Xerostomia severity was quantified by the Xerostomia Inventory (XI). Consumed anti-hypertensive drugs, age and gender were also recorded. Data were analyzed using SPSS 22 (IBM version 22.0, Chicago, USA) and statistical significance level was considered P0.05.

**Results:**

The mean age of participants was score of OHIP-14 was 58.90 ± 5.66 years, of whom 53% were male and 47% female. The mean total OHIP-14 score was 13.33 ± 11.11. Patients with xerostomia consisted 78% of the sample, with a mean XI score of 23.9 ± 5.1. Total and all domain scores of OHIP-14 were directly and significantly correlated with XI score (P0.001). Also, age had a direct and significant correlation with both OHIP-14 (P0.001) and XI (P=0.001) scores. Use of cardiovascular drugs was not significantly related to xerostomia presence or severity (P0.05).

**Conclusion:**

The majority of cardiovascular patients suffer from moderate xerostomia, regardless to the type of medication they consume. Xerostomia affects OHRQoL in these patients and should be treated properly to improve their life quality, especially among the elderly.

## Introduction

Oral health related quality of life (OHRQoL) defines the effect of oral health status on several aspects of daily life. In other words, OHRQoL measures whether oral functions such as eating, speaking or esthetics are qualified enough to satisfy patient’s physical, emotional and psychological needs, or not [1]. The interference of oral health problems with one’s life is assessed subjectively and its treatment is considered as the final goal of objective oral therapies [2]. Oral Health Impact Profile 14 (OHIP-14) questionnaire is the most applied means to quantify OHRQoL [3].

The subjective feeling of dry mouth is called xerostomia, Hyposalivation may or may not co-exist with such a feeling [4]. Several factors may induce such a perception, including advanced age, dehydration, oral respiration, and cigarette, alcohol or drug consumption [5]. The feeling usually urges patient to sip water frequently. Several oral complications have been reported to accompany xerostomia, such as burning mouth, candidiasis, pain and difficulty in chewing, speaking or swallowing, and halitosis [6]. As a subjective symptom, xerostomia presence or severity can be assessed by some developed questionnaires [7].

Cardiovascular diseases encompass a wide range of morbidities and the patients typically take several medications, due to hypertension, ischemic heart disease, arrhythmia or heart failure [8][9]. Such a poly-pharmacy condition may induce xerostomia, especially in the elderly, putting them at risk for miscellaneous oral side effects [10][11]. In turn, these effects may predispose OHRQoL deterioration by threatening patient physical, mental or oral health [12]. Therefore, the aim of this study was to evaluate OHRQoL and its relation to xerostomia and consumed drugs in cardiac patients.

## Materials and Methods

### Patients and Sampling

This was a cross-sectional study. Outpatients with hypertension, ischemic heart disease or heart failure were invited to participate. Inclusion criterion was consuming any type of cardiovascular drugs. Exclusion criteria consisted of the conditions that confound salivary function, such as anorexia, bulimia, chronic alcoholism, Sjogren’s syndrome, tuberculosis, sarcoidosis, Crohn’s disease, Wegener’s granulomatosis, as well as previous chemotherapy/head and neck radiotherapy.

Convenient sampling was used. With a 95% confidence, a sample size of 244 patients was calculated. Patients attending Isfahan University of Medical Science Cardiovascular Clinic for their routine follow-ups were invited by the researcher, and after informed consent, participated the present study.

### Data Collection

Data were collected by several means. OHIP-14 was used to assess OHRQoL. It is valid and reliable for Persian natives [13]. It consists of 14 questions in 7 domains. Each answer was scored by a Likert scale, with 0 for "never" to 4 for "almost always". Therefore, total OHIP-14 score ranged from 0 to 56, with higher scores speaking for worse OHRQoL.

Two scales were used to assess xerostomia. To explore xerostomia, ten questions were asked from each participant. Positive answer to 3 or more questions set him/her as xerostomia positive [14]. Afterwards, patients with xerostomia were asked to fill out the Xerostomia Inventory (XI) index, which contains 11 questions, valid and reliable for Persian language [15]. Each question had a score of 1 (never) to 5 (almost always) in a Likert scale. Therefore, the total XI score ranged between 11 and 55, with higher scores representing worse xerostomia severity. Consumed blood pressure lowering drugs were categorized and recorded as diuretics, beta-blockers, angiotensin converting enzyme (ACE) inhibitors and angiotensin receptor blockers (ARB), using patients’ medical record [16][17]. Patient gender and age were also recorded.

### Statistical Analyses

SPSS version 22 was used to analyze data. Chi square, T test and Spearman’s/Pearson’s correlation coefficients were used. P<0.05 was considered statistically significant.

### Ehical Considerations

Local ethics committee passed the study protocol (ethical code: IR.MUI.RESEARCH.REC.1400.124). Not willing to participate did not affect the patient’s planned treatment.

## Results

**Table:** Table1. Mean Scores of OHIP-14 Domains and Their Correlation with XI Score

**Domain**	**Mean (± SD)**	***P*** **value**	**Spearman’s *r* [95% CI] **
Functional limitation	1.51 (± 1.75)	<0.001 ^*^	0.698 [0.593, 0.787]
Physical pain	1.45 (± 1.69)	<0.001 ^*^	0.656 [0.554, 0.748]
Psychological discomfort	1.70 (± 1.75)	<0.001 ^*^	0.643 [0.561, 0.741]
Physical disability	1.98 (± 1.95)	<0.001 ^*^	0.642 [0.528, 0.777]
Psychological disability	1.96 (± 1.84)	<0.001 ^*^	0.703 [0.621, 0.779]
Social disability	2.26 (± 1.64)	<0.001 ^*^	0.578 [0.495, 0.603]
Handicap	2.46 (± 1.68)	<0.001 ^*^	0.616 [0.560, 0.681]
Total	13.33 (± 11.11)	<0.001 ^*^	0.722 [0.669, 0.770]

OHIP: Oral Health Impact Profile, XI: Xerostomia Inventory, SD: Standard Deviation, CI: Confidence Interval ^*^statistically significant

**Table:** Table2. Mean Age and OHIP-14 Scores in Patients with/without Xerostomia

**Variable (mean)**	**Xerostomia**		**P Value**	**Mean Difference [95% CI]**
	**Yes**	**No**		
**Age (years)**	60.96	50.44	0.022 ^*^	10.52 [8.80, 17.31]
**OHIP-14 (score)**	16.41	3.14	0.001 ^*^	13.27 [8.99, 26.08]

OHIP: Oral Health Impact Profile, CI: Confidence Interval ^*^Statistically significant

**Figure-1:**
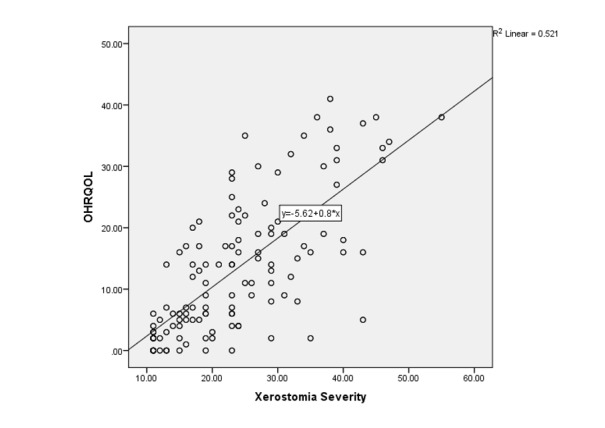
Figure 1. The correlation between xerostomia severity and oral health related quality of life (OHRQoL)

The sample consisted of 244 patients, 130 (53%) male and 114 (47%) female, with a mean age of 58.90 ± 5.66 years old. One hundred and ninety patients (78%) were xerostomia positive, with a mean XI score of 23.9 ± 5.1. Beta blockers, diuretics, ARB and ACE inhibitors were consumed by 76.2%, 48.4%, 38.5% and 32% of patients, respectively. Also, mean total OHIP-14 score was 13.33 ± 11.11.

Table-1 shows domain scores of OHIP-14 (Table-1). Pearson’s correlation coefficient test showed that increasing age was significantly correlated with total OHIP-14 Score (P<0.001, r=0.715 [0.660, 0.772]) and XI score (P=0.001, r=0.621 [0.530, 0.700]). Besides, total and all domain scores of OHIP-14 were significantly correlated with XI score (Figure-1).

T-test showed that patients with xerostomia were significantly older and had a worse OHRQoL than patients without this morbidity (Table-2). Meanwhile, there was no significant difference between XI or OHIP-14 scores between men and women (P>0.05). Chi square test revealed no significant relationship between xerostomia presence and gender either (P=0.704).

Of note, there was no significant relationship between diuretic (P=0.31), beta-blocker (P=0.16), ACE inhibitor (P=0.18) or ARB drug consumption (P=0.07) and xerostomia presence. There was no significant relationship between diuretic (P=0.36), beta-blocker (P=0.79), ACE inhibitor (P=0.47) or ARB drug consumption (P=0.68) and xerostomia severity either. Multiple linear regression analysis showed that age and xerostomia severity were significantly related to OHRQoL (P=0.001 and P<0.001, respectively).

## Discussion

OHRQoL is a multi-dimensional phenomenon affected by a web of health variables, including physical, psychological and oral well-being [18]. Cardiovascular diseases may influence OHRQoL directly by deteriorating physical conditions related to oral functions, such as eating or speaking. On the other hand, they may exhaust the patient in a way that he/she ignores local oral health behaviors, leading to a mental suffer due to the oral condition [12].

Schmalz et al. in their 2021 study evaluated OHRQoL, by OHIP-14, in heart failure patients (5.35±2.92) and those with Left Ventricular Assist Device (LVAD) (6.82±3.53). They obviously reported less OHIP-14 scores than the present study (13.33±11.11). It seems that better health support services in Germany could have improved OHRQoL among patients with advanced cardiovascular morbidity [19].

Molania et al. assessed OHIP-14 among 240 Iranian cardiovascular patients, with a mean age of 59.34±1.18 years, in 2020. Despite the similarity of the patient sample between their study and this research, they reported a quite higher mean questionnaire score of 21.34±17.04, in comparison with 13.33±11.11. More attention towards Iranian cardiovascular patients seems vital to ameliorate their OHRQoL condition [18].

In accordance with the present results, several studies have proved the impact of xerostomia on OHRQoL, both in medically compromised patients and the general population. Molania et al. and Chamani et al. proved this impact in their 2017 studies among diabetics and rheumatoid arthritis patients, respectively [20][21]. Ahmad et al. and Niklander et al. came across the same results among general population in 2017 too [2][22].

Parat et al. and Schmalz et al., in their 2020 studies, depicted significant relationship between poor oral health indices and low OHRQoL in scleroderma and cardiovascular patients, respectively [23][24]. Xerostomia can leave the dentition defenseless against cariogenic bacteria and predisposes rampant caries. Besides, lack of oral lubrication affects speech directly and probably.

The present study showed significant correlation between XI and OHIP-14 scores (P<0.001). This fact is in accordance with Molania et al. study [18] and urges preparation of prompt services to improve cardiovascular patients’ OHRQoL as a consequence of oral health condition. In line, the study by Ingolotti et al. in 2025 confirmed the impact of xerostomia on OHRQoL in Sjogren’s syndrome patients [25]. Moreover, multiple regression analysis in the present study revealed another significant contributor to OHRQoL in these patients.

Irrespective to the several consumed drug types, both XI and OHIP-14 scores were significantly correlated with age (P=0.001 and P<0.001, respectively). Old patients may be engaged by miscellaneous systemic diseases simultaneously. It seems that lack of attention and support, rather than any special anti-hypertensive drug, aggressively exacerbates oral health and OHRQoL. This fact is further confirmed by the study of Lim et al. in 2025, which evaluated OHRQoL in cardiovascular patients as well [26]. General health is a known factor in determining OHRQoL [1]. Although the present cross-sectional study has the limitation of inability to explore a cause-effect relationship, it found the strong impact of aging on OHRQoL. Future longitudinal studies are recommended to track causal relationships between xerostomia and OHRQoL over time. Aging probably reflects lack of sufficient financial and psychological back-ups, as well as presence of other comorbidities and systemic diseases (e.g. diabetes). Future studies might better elucidate the reciprocal effect of such variables on OHRQoL in cardiovascular patients.

The present study relied on a single cardiovascular clinic, and the sampling method was convenient. It is recommended to carry out multi-center studies in future to improve result generalizability. Besides, the present study does not compare the results with a control group. Such a comparison in future studies may better differentiate the impact of cardiovascular disease from other causes of xerostomia. Also, objective salivary flow rate assessment (e.g. sialometry) in future studies can confirm hyposalivation and minimize bias.

## Conclusion

Presence and severity of xerostomia, as well as increasing age, were major determinants of OHRQoL in cardiovascular patients. Prioritizing treatments to relief xerostomia and its comorbidities, along with paying attention to social, financial and psychological supports are recommended in order to upgrade the level of life quality among these patients, specifically their older population.

## Conflicts of Interest

The authors of this manuscript declare that they have no conflicts of interest, real or perceived, and financial or non-financial in this article.
